# Investigating the contribution of IL-17A and IL-17F to the host response during *Escherichia coli* mastitis

**DOI:** 10.1186/s13567-015-0201-4

**Published:** 2015-06-11

**Authors:** Perrine Roussel, Patricia Cunha, Adeline Porcherie, Wolfram Petzl, Florence B Gilbert, Céline Riollet, Holm Zerbe, Pascal Rainard, Pierre Germon

**Affiliations:** INRA, UMR1282, Infectiologie et Santé Publique, F-37380 Nouzilly, France; Université François Rabelais de Tours, UMR1282, Infectiologie et Santé Publique, F-37000 Tours, France; Clinic for Ruminants with Ambulatory and Herd Health Services at the Centre for Clinical Veterinary Medicine, Ludwig-Maximilians University Munich, Oberschleissheim, Germany

## Abstract

Mastitis remains a major disease of cattle with a strong impact on the dairy industry. There is a growing interest in understanding how cell mediated immunity contributes to the defence of the mammary gland against invading mastitis causing bacteria. Cytokines belonging to the IL-17 family, and the cells that produce them, have been described as important modulators of the innate immunity, in particular that of epithelial cells. We report here that expression of IL-17A and IL-17F genes, encoding two members of the IL-17 family, are induced in udder tissues of cows experimentally infected with *Escherichia coli*. The impact of IL-17A on the innate response of bovine mammary epithelial cells was investigated using a newly isolated cell line, the PS cell line. We first showed that PS cells, similar to primary bovine mammary epithelial cells, were able to respond to agonists of TLR2 and to LPS, provided CD14 was added to the culture medium. We then showed that secretion of CXCL8 and transcription of innate immunity related-genes by PS cells were increased by IL-17A, in particular when these cells were stimulated with live *E. coli* bacteria. Together with data from the literature, these results support the hypothesis that IL-17A and IL-17 F could play an important role in mediating of host-pathogen interactions during mastitis.

**Electronic supplementary material:**

The online version of this article (doi:10.1186/s13567-015-0201-4) contains supplementary material, which is available to authorized users.

## Introduction

Despite decades of research, mastitis remains a major concern in dairy farming. Mastitis are mainly due to bacterial infections (Gram-positive pathogens such as *Staphylococcus aureus* and *Streptococcus uberis*, or Gram-negative pathogens such as *Escherichia coli*) [1].

Previous studies have allowed the identification of key molecular events that ultimately lead to the recruitment of neutrophils in the mammary gland upon bacterial colonization. When bacteria enter the udder, the host responds by secreting chemokines (such as CXCL8, CCL20) and other immune components such as antimicrobial peptides (LAP—lingual antimicrobial peptide, TAP—tracheal antimicrobial peptide) and pro-inflammatory cytokines (TNF-α, IL-1β) [2-4]. Production of pro-inflammatory cytokines such as IL-1β, IL-6 and TNF-α and the chemokine CXCL8 have been detected in milk from clinically affected animals and are supposed to contribute to the inflammation observed in mastitis [2,4].

In vivo experiments indicate that the severity of *E. coli* mastitis mainly depends on host factors and that a quick and efficient response is important for an efficient clearance of the bacteria [5]. This process relies heavily on the recruitment of neutrophils during infection: a delay in the recruitment of neutrophils aggravates the infection [6,7]. It is therefore expected that any mechanism that modulates the immune response of the host could participate in the defence against *E. coli* mastitis.

IL-17A and IL-17F are two cytokines that have been described as playing a significant role in the recuitment of neutrophils in other inflammatory diseases. Along with four other structurally related cytokines, IL-17B, IL-17C, IL-17D and IL-17E, they form the IL-17 family [8].

Although expression of IL-17A and IL-17F may be detrimental to the host, in particular in the case of autoimmune diseases, they have been shown to be beneficial to the host to fight against bacterial pathogens such as *Citrobacter rodentium*, *Klebsiella pneumoniae* or *Escherichia coli* [8,9].

Production of IL-17A during *S. uberis* mastitis was recently demonstrated [10]. Tao and Mallard also reported that IL-17A gene expression was slightly increased (approx. 1.5-fold in milk) in somatic cells from *S. aureus* infected cows [11]. Microarray analyses of MEC stimulated with *S. aureus* culture supernatant also showed induction of the IL-17A pro-inflammatory pathway [12].

In addition, we recently demonstrated that, in vitro, IL-17A increases the ability of mammary epithelial cells (MEC) to respond to agonists similar to that produced by *S. aureus* [13]. These cells are thought to play a significant role in the defence against invading pathogens by producing antimicrobial peptides as well as cytokines and chemokines such as CXCL8 and IL-6. Indeed, in vitro grown primary bovine MEC (pbMEC) have been shown to respond to the presence of bacteria, such as *E. coli* or *S. aureus*, or to different purified bacterial agonists by producing different cytokines, chemokines or antimicrobial peptides [12,14-18].

Based on these data, one could hypothesize that IL-17 cytokines contribute to the innate response of the host during *E. coli* mastitis; but this remains to be studied.

In the present report, we thus decided to investigate, under controlled conditions, whether expression of genes encoding cytokines of the IL-17 family was induced upon intra-mammary infection of cows by *E. coli*. We then explored how this over-expression of IL-17A gene could translate in terms of defence against the invading pathogen. Using a new cell line, called the PS cell line, which we characterized in details in terms of phenotypic markers and response to bacterial agonists, we analysed the influence of IL-17A on the innate immune response of MEC to infection by *E. coli*.

This report brings a new perspective on the cellular features and molecular parameters involved in the ability of MEC to take part in the udder defence against mastitis.

## Materials and methods

### Ethics statement

Experimental infection of animals were conducted at the Clinic for Ruminants (Munich, Germany) with the approval of the ethics committee of the regional government of upper Bavaria, Germany (No. 55.2-1-54-2531-108-05).

### Experimental infection of cows

Five healthy German Holstein Frisian first lactation cows in mid lactation (3-6 months after parturition) fulfilling criteria as previously described [19] were inoculated in one quarter with 500 cfu *E. coli* strain 1303. Five heifers that received no treatment served as untreated controls. Only animals without previous diagnosis of clinical or subclinical mastitis and a reported somatic cell count <50 000/mL were included in the study. Quarter milk samples were collected and tested weekly before the trial to ensure that they contained <50 000 somatic cells/mL and were free of mastitis pathogens. Animals were randomly assigned to both groups and the experiments were carried out between March and December. All *E. coli* inoculated animals developed clinical mastitis in the affected quarter 12 h after inoculation as previously described [19]*.* Animals were slaughtered 24 hours post-inoculation (hpi). Liquid nitrogen snap frozen udder samples of lobulo-alveolar tissue 7 cm dorsal of the milk cistern were obtained immediately after slaughtering. RNA was isolated from approx. 100 mg of frozen udder tissue using Trizol (Invitrogen). The sample was placed in a 2 mL tube containing 1.4 mm beads (MP Biomedicals) and one mL of Trizol was added. Tissue lysis was obtained by shaking the tubes twice in a FastPrep apparatus (MP Biomedicals) for 45 s at speed 6. The homogenate was further processed as recommended by the manufacturer. RNA quality was checked using an Agilent Bioanalyzer and only samples with a RNA Integrity number above 7 were used. Controls included RNA samples from the uninoculated quarters from inoculated cows as well as samples from quarters of non-inoculated cows.

### Isolation and culture of PS cells

The whole mammary gland was isolated from a Prim’Holstein dairy cow. The cow was killed at the slaughterhouse of the INRA dairy facility as part of a routine killing at the end of its 6^th^ lactation. The cow was killed following the recommended guidelines of the American Veterinary Medical Association (“AMVA Guidelines for the Euthanasia of Animals”): first the cow was euthanized using a penetrating captive bolt and killed by exsanguination by the authorized personnel of the slaughterhouse. The mammary gland was removed and transferred to the laboratory for further processing. Pieces of tissue were dissected in the secretory parenchyma and placed in Hank’s Balanced Salt Solution (HBSS, Lonza) containing 200 U/mL penicillin (Sigma-Aldrich), 200 μg/mL streptomycin (Sigma-Aldrich), 0.5 μg/mL amphotericin B (Gibco). Tissue fragments of 10 g were washed with HBSS and minced with surgical scissors. The small tissue cubes were washed and incubated at 37 °C under slight agitation in 30 mL HBSS containing 5.5 U/mL protease from *Streptomyces griseus* (Sigma-Aldrich). Every 30 min, the dispersed cells were harvested from the mixture by filtration through a 200 μm nylon mesh and fresh enzymatic preparation was added to the remaining tissue fragments. Cells used in this study were obtained after 2 h of incubation with pronase. Cells were cultured at 37 °C in 5% CO_2_ in Advanced DMEM/F12 medium (Gibco) containing 10% fetal calf serum (FCS), 2 mM L-glutamine, 40 mM *N*-(2-hydroxyethyl)-piperazine-*N*′-2-ethanesulfonic acid (HEPES buffer, Biowhittaker), 200 U/mL penicillin (Sigma-Aldrich), 200 μg/mL streptomycin (Sigma-Aldrich), 0.5 μg/mL amphotericin B (Gibco) and 1 μg/mL hydrocortisone (Sigma-Aldrich). After 24 h, attached cells were washed with HBSS and fresh medium was added. After 3 days of culture, epithelial cells were enriched by selective trypsinization (0.25% trysin-EDTA). Detached cells after 5 min of trypsinization were discarded and fresh medium was added on the cells that remained attached to the plastic of the culture flasks. This selective trypsinization was repeated on day 6 and a first passage was performed two days later. Cells were then frozen under nitrogen after 2 passages. The PS cell line was obtained by selective trypsinization at passage 4. The cells were subjected to 2 additional passages and frozen.

Cells were thereafter maintained in MEC growth medium (GM): Advanced-DMEM/F12 (Gibco) containing 20 mM HEPES, 2 mM L-glutamine (Gibco), 1 μg/mL hydrocortisone (Sigma-Aldrich), 10 ng/mL Insulin-like growth factor (IGF)-1, 5 ng/mL Fibroblast growth factor (FGF), 5 ng/mL Epidermal growth factor (EGF).

### Cytology of the PS cell line

In order to visualize the morphological features of the PS cell line in culture, a May-Grünwald Giemsa staining was performed on cells grown on 6-well plate. Cells were covered with May-Grünwald dye for 3 min. The same volume of distilled water was added for 1 min. After draining, Giemsa dye (8% in distilled water) was incubated for 20 min and the cells were washed with tap water.

### Flow cytometry analysis of PS cells

Antibodies used in the present study are described in Additional file 1. Labellings were done on PS cells at passages 9, 14 and 18. Intracellular labelling for cytokeratins expression study was performed when cells formed a confluent monolayer. Cells were harvested after trypsin treatment (0.05% trysin-EDTA -Gibco), and the cell pellet was resuspended in 500 μL of permeabilization buffer Cytofix Cytoperm (BD Biosciences), and incubated in the dark at 4 °C. After 20 min, the cell suspension was washed with 1 mL of Perm Wash 1X buffer (BD Biosciences). The cells were collected, resuspended in Perm Wash 1X buffer, and were split into each well (3 × 10^5^/well in 20 μL) of a 96-well U-bottom well plate. The cells were then incubated with 20 μL of primary antibody for 15 min at 4 °C. The plate was washed with 100 μL of Perm Wash buffer, and the cell pellet was resuspended in 50 μL of the secondary antibody for 15 min at 4 °C when required.

Extracellular labelling was performed when MEC formed a confluent monolayer. Cells were harvested and distributed (20 μL, 3 × 10^5^ cells/well) into 96-well U-bottom well plates. The cell suspension was resuspended in 20 μL of antibodies against CD45, CD14 and CD282 (TLR2), and incubated for 15 min at 4 °C. The cells were then washed with 100 μL of buffer (DPBS without Ca and Mg, Lonza; 2% goat serum, Gibco; 2 mM EDTA). The plate was centrifuged for 10 min at 200 *g*, and, if necessary, the cell pellet was resuspended in 50 μL of secondary antibodies, and incubated for 15 min at 4 °C. Then the cells were washed and fixed with 150 μL of FACS lysing solution (BD Biosciences). Peripheral blood mononuclear cells (PBMC) isolated from bovine blood were used as positive control for CD45 labelling. Fluorescence-activated cell sorting (FACS) analysis was performed on a FACSCalibur cytometer using CellQuest software (BD Biosciences) and data were analyzed with FlowJo software (Tree Star, Ashland, OR, USA).

### Stimulation of cells with live bacteria or purified bacterial agonists

For each stimulation assay, each stimulus/strain was assessed in duplicate wells. PS cells were seeded at a concentration of 10^5^ cells/mL in 24-well or 96-well plates and cultivated in GM medium until they formed a confluent monolayer. They were then cultured overnight (16 h) in fresh stimulating medium without growth factors (stimulation medium). Cells were then washed twice with HBSS and stimulated with either live bacteria or bacterial purified agonists diluted in stimulation medium at the indicated concentrations. For stimulation with *E. coli* strains 1303 or P4 [20], bacteria were grown overnight in stimulation medium at 37 °C without agitation. On the day of the experiment, bacteria were diluted in stimulation medium and added to cells at a multiplicity of infection of 1. Cells were incubated with bacteria for three hours at 37 °C in a humidified incubator with 5% CO_2_; bacteria were then removed, cells were washed twice with HBSS and stimulation medium containing 10 μg/mL gentamycin was added. Supernatant was collected 5 h or 21 h after addition of the gentamycin containing medium and cells were processed for RNA purification if necessary (see below). Agonists used were from InvivoGen: ultrapure lipopolysaccharide from *E. coli* O111:B4 strain (LPS), γ-D-glutamyl-meso-diaminopimelic acid (C12-iE-DAP), Pam2-CSK4, Pam3-CSK4, muramyl-dipeptide (MDP) and flagellin (FLA-ST Ultrapure). Cells were incubated with these purified agonists for 5 h at 37 °C in a humidified incubator with 5% CO_2_; supernatants were then collected and cells were processed for RNA purification if necessary. For RNA isolation, cells were lysed with RA1 lysing buffer (Macherey-Nagel) with 1% β-mercaptoethanol (Sigma-Aldrich). RNA was then extracted following the manufacturer’s instructions (Macherey-Nagel). When required, 5 μg/mL of recombinant human soluble CD14 (CD14) (Biometec GmbH, Germany), 5 μg/mL of recombinant human LPS-binding protein (LBP) (Biometec GmbH) or fresh whole milk were used to supplement the culture medium of PS cells. Fresh whole milk was collected on the day of the experiment from a healthy Prim’Holstein cow from the Unité Expérimentale de Physiologie Animale de l’Orfrasière (INRA Tours). IL-17A was used as described previously at a concentration of 100 ng/mL [13]. Primary cells were isolated and grown as previously described and used at their third passage [16,21,22].

### ELISA assays

CXCL8, IL-17A and TNFα production by PS cells was quantified by sandwich ELISA, with reference to standard as described previously [23,24]. IL-1β was quantified by ELISA using the Bovine IL-1 beta ELISA Reagent Kit (Thermo Scientific).

### Transcript analysis by RT-PCR and RT-qPCR

Total RNA (1 μg) was reverse transcribed to cDNA using random hexamers and SuperScript RT III (Invitrogen) according to manufacturer’s instructions. Primers used in this study are listed in Additional file 2 and [13]. Expression of PRRs was analyzed by RT-PCR using 40 amplification cycles. Relative quantities of transcripts of interest were analyzed by RT-qPCR as described previously [13]. Briefly, after normalization using the expression of 3 reference genes (Actb, PPIA, 18S RNA), expression of each gene was calculated relative to the values obtained from unstimulated cells arbitrarily set to 1.

### Statistical analysis

In vivo gene expression results were analysed by a permutation test using the StaXact software (v5.0—Cytel software) to obtain exact *p*-values. For the analysis of responses of MEC cells to the different bacterial agonists or to live bacteria, data were first compared using the Kruskal and Wallis test followed by exact permutation tests using StatXact. A *p*-value < 0.05 was considered significant.

## Results

### IL-17A and IL-17 F gene expression is induced upon infection of the udder by *E. coli*

**Figure 1:**
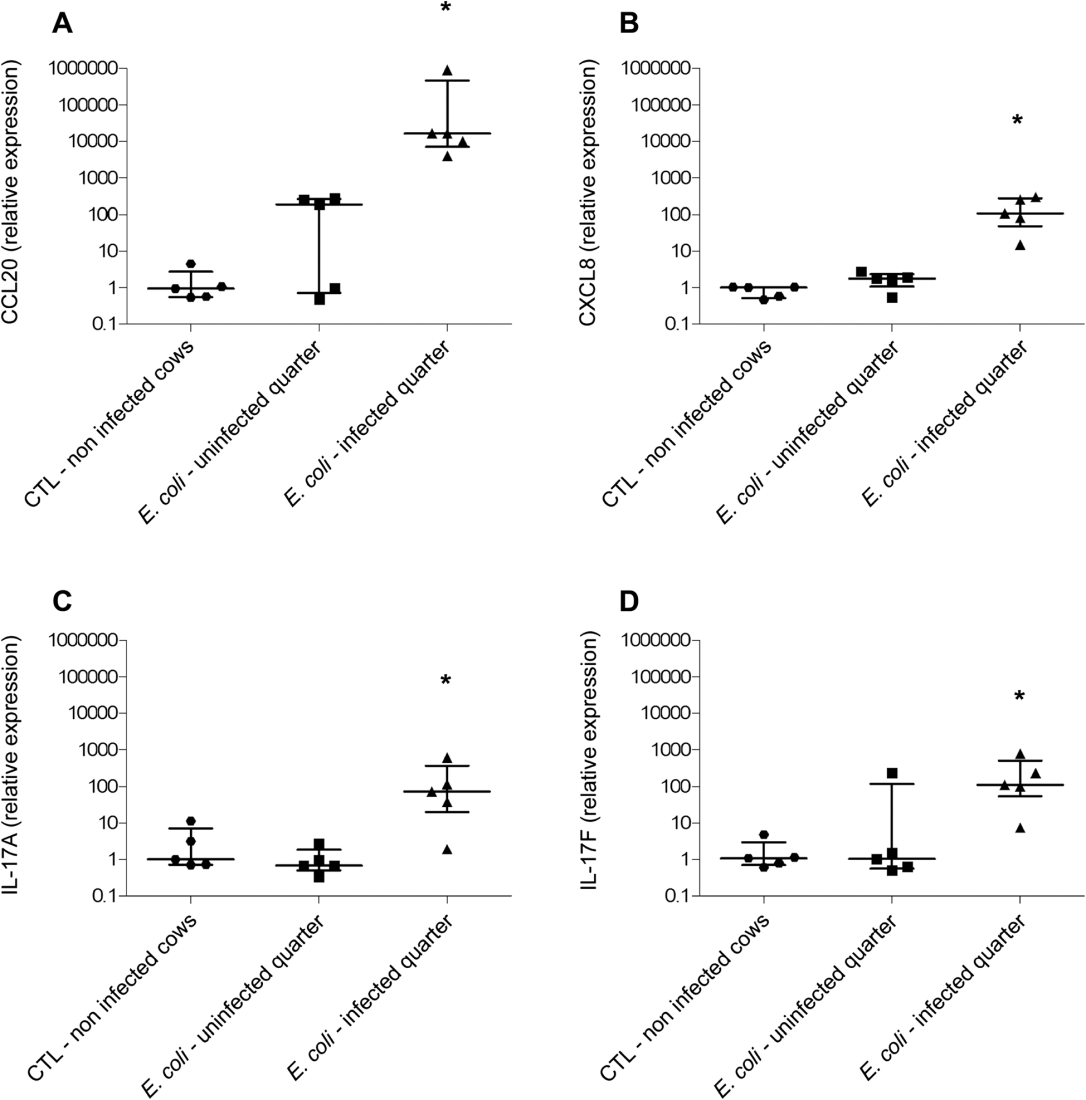
**Analysis of gene expression during experimentally induced mastitis.** RT-qPCR specific for CCL20 (**A**), CXCL8 (**B**), IL-17A (**C**) and IL-17F (**D**) were performed on RNA extracted from tissue samples of udder quarters from uninfected cows or *E. coli* infected cows. Data presented are values from five animals with medians and interquartile ranges indicated as vertical bars. * statistical significance (*P* < 0.05) of infected versus uninfected cows calculated using exact permutation tests after global comparison using a Kruskal and Wallis test.

Previous reports showed that experimental intra-mammary inoculation of *Escherichia coli* 1303 induced a significant host response [25]. Based on microarray analyses, expression of cytokines and chemokines such as CCL20 and CXCL8 was shown to be increased upon *E. coli* 1303 infection 514 and 58-fold, respectively [15]. Our RT-qPCR data confirmed that, upon infection with *E. coli* 1303, expressions of CCL20 and CXCL8 genes were induced, approx. 16 000-fold and 108-fold, respectively (Figures 1A and B). A higher expression, compared to uninfected cows, was also detected, particularly with CCL20, in uninfected quarters of three out of five *E. coli* infected cows which is consistent with a systemic effect as previously reported [19,26].

Microarray experiments had failed to detect a significant expression of IL-17 family genes in udder tissue after *E. coli* infection, presumably because of a low expression of these genes [15]. In the present study, we thus used RT-qPCR to evaluate the expression of IL-17 family genes. No expression was detected for IL-17C and IL-17E (data not shown). On the contrary, expression of IL-17A and IL-17F was significantly increased in *E. coli* infected quarters, compared to non-infected quarters (Figures 1C and D): the mean fold-change for IL-17A was 72 in samples from cows infected by *E. coli*. Mean induction of IL-17F was 109-fold in the same samples (Figures 1C and D). Although induction of IL-17A gene was clearly observed in our tissue samples, IL-17A levels in milk samples from *E. coli* infected cows were below the detection limit of the ELISA assay used, that is below 200 pg/mL in milk.

Despite this lack of detection of IL-17A in milk from infected quarters, gene expression results suggest that IL-17A and IL-17F may play a role in the immune response of cows to intra-mammary infection, either at the onset of or during the inflammatory response.

Considering that IL-17A has generally more potent effects than IL-17F on epithelial cells [13], we then explored if IL-17A in particular could contribute to the defence of the host against *E. coli* infection by modulating the innate immune response of MEC. Indeed, these cells are key players in mounting an efficient early innate immune response and IL17-A has been shown to target a variety of cell types, in particular epithelial cells [27-29].

### PS cells as a useful tool to study the innate immune response of mammary epithelial cells

Although pbMEC are often used to study the response of MEC to infection, the use of primary epithelial cell cultures has a number of drawbacks. Most importantly, variability of response of pbMEC from different animals on the one hand and lack of reproducibility of experiments on the other hand, can be associated with contamination of primary cell cultures with other cell types, and the amount of these contaminating cells can vary from one primary culture to another. Considering that these contaminating cells could exacerbate or lower the innate immune response of MEC through cooperation, we decided to use a new mammary epithelial cell line showing innate immune response capabilities to perform these assays.

**Figure 2:**
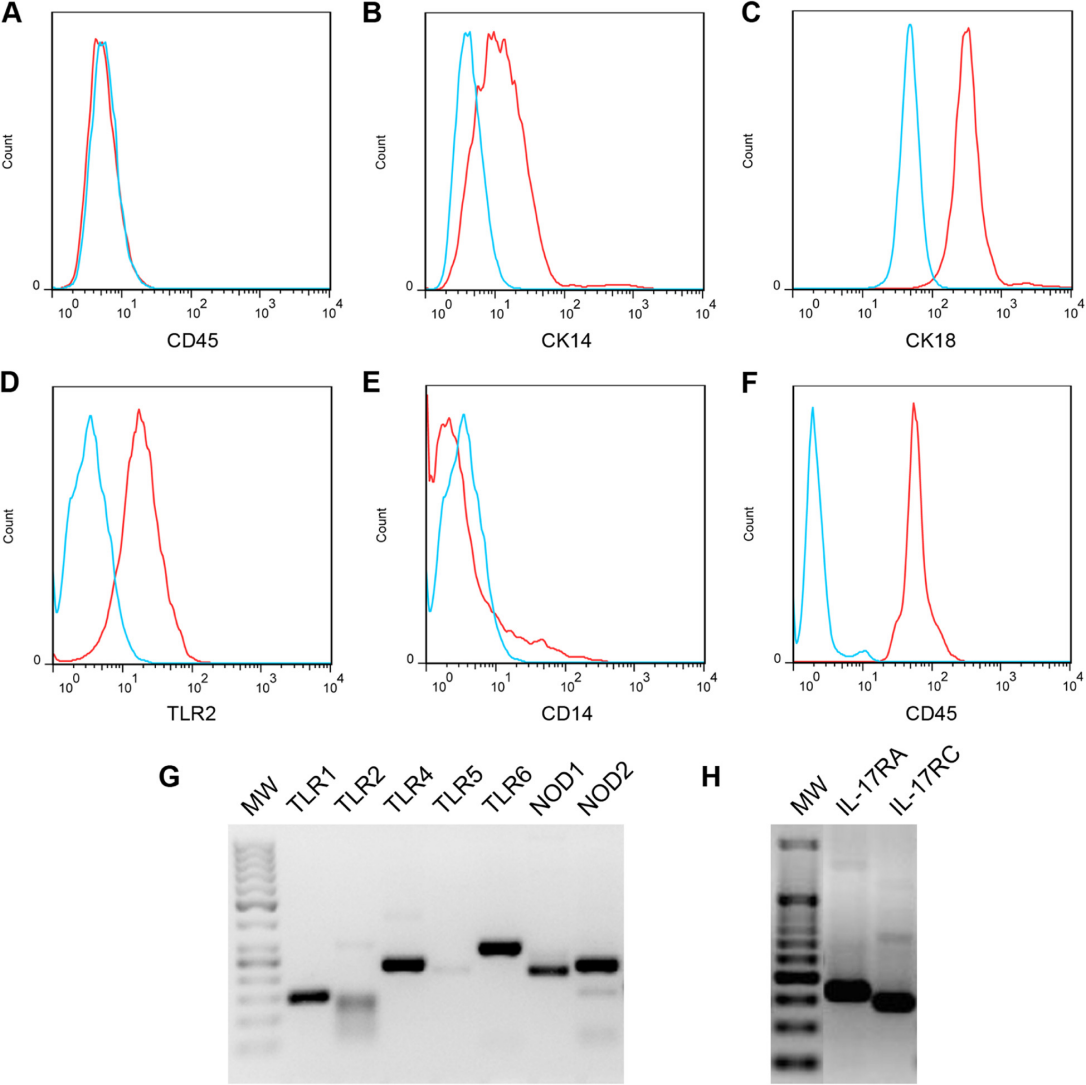
**Characterization of PS cells properties by flow cytometry and RT-PCR.** PS cells, at passage 14, were labelled with antibodies against CD45 (**A**), cytokeratin 14 (**B**), cytokeratin 18 (**C**), TLR2 (**D**) and CD14 (**E**). Controls were performed either by omitting the primary antibody (**A**, **B**, **C**) or using unlabelled cells (**D**, **E**). As a control for CD45 labelling, bovine PBMC were labelled with the anti-CD45 antibody (**F**). Labelled cells are depicted as red curves while controls are represented as blue curves. (**G**-**H**) RNA was extracted from unstimulated PS cells and converted to cDNA. cDNAs for each of the indicated receptors were amplified by PCR and products were separated by agarose gel electrophoresis. MW: 50 bp molecular weight ladder.

This cell line, which we called the PS cell line, stems from spontaneously immortalized cells that emerged from a culture of primary bovine MEC isolated from the *s*ecretory *p*arenchyma of a cow (hence the name PS cells). Upon reaching confluency, they form a homogeneous layer of cells with cobblestone morphology typical of epithelial cells (Additional file 3). This cell line was also characterized for potential contamination by leukocytes (CD45-positive cells) and for expression of cell surface markers associated with mammary epithelial cells. Results indicated a percentage of CD45-positive cells similar to background level (0.6%) and expression of cytokeratin 18 by more than 99.5% of cells. Cytokeratin 14 was only weakly expressed by PS cells (Figures 2A-F).

RT-PCR data indicated that these cells expressed several genes encoding Pattern Recognition Receptors (PRR) such as TLR1, TLR2, TLR4, TLR6, NOD1 and NOD2 (Figure 2G) as well as the genes encoding the two subunits of the IL-17A receptor, namely IL-17RA and IL-17RC (Figure 2H). While the weak amplification of TLR5 cDNA was consistent with previous results obtained with primary epithelial cells [16], a weak amplification of TLR2 cDNA was observed. Despite this low expression of TLR2, FACS analysis showed that the TLR2 protein was actually present at the surface of PS cells (Figure 2D).

**Figure 3:**
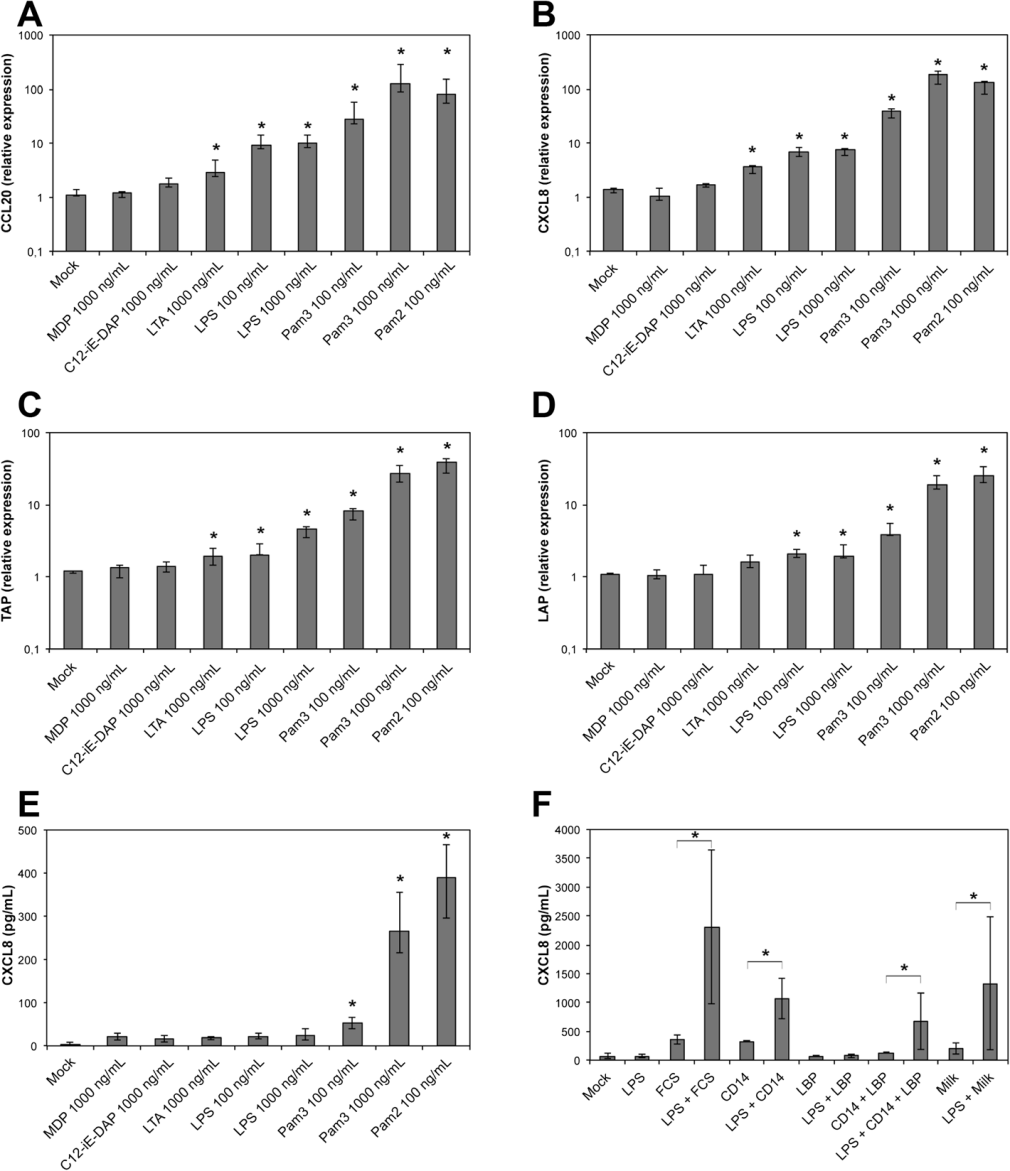
**Innate immune response of PS cells to different purified bacterial agonists. PS cells were incubated for 5 h with the indicated concentrations of different purified agonists.** Response was analyzed in terms of expression of CCL20 (**A**), CXCL8 (**B**), TAP (**C**), LAP (**D**) genes by RT-qPCR or CXCL8 secretion by ELISA (**E**,**F**). Data presented are mean values and SD obtained from 3 independent experiments. Experiments were performed at passages 11, 12 and 24. PS cells were incubated for 5 hours in GM medium with different agonists at the indicated concentrations. (F) PS cells were incubated with ultrapure LPS (LPS) at 10 ng/mL in the presence of either 10% fetal calf serum, 5 μg/mL CD14 or 5 μg/mL LBP. Stimulation was also performed in whole milk instead of GM medium. * statistical significance (*P* < 0.05) of stimulated versus unstimulated PS cells using exact permutation tests after global comparison using a Kruskal and Wallis test.

In order to use these cells to study the innate immune response, we analysed their capacity to respond to stimuli such as lipopolysaccharide (LPS), lipoteichoic acid (LTA), lipoproteins or peptidoglycan degradation products. The synthetic molecules Pam2-CSK4 and Pam3-CSK4 are analogs of di-acylated and tri-acylated lipoproteins that signal through the TLR2/TLR6 or TLR1/TLR2 heterodimers, respectively. C12-iE-DAP and MDP are peptidoglycan subunits that are sensed by the NOD1 and NOD2 receptors, respectively. When PS cells were exposed to these MAMPs, the highest response was observed with Pam2-CSK4 at 100 ng/mL (Figure 3): compared to non-induced cells, Pam2-CSK4 induced an overexpression of CCL20, CXCL8 and TAP, three genes whose expression is induced upon stimulation of mammary epithelial cells [15], and induced also the secretion of CXCL8 (Figure 3E). PS cells also responded to Pam2-CSK4 when used at 10 ng/mL (data not shown). A significant but lower response was observed with Pam3-CSK4, both in terms of increased expression of CCL20, CXCL8, TAP and LAP and increased secretion of CXCL8 (Figure 3). No response was observed with both MDP and C12-iE-DAP (Figure 3). In a separate experiment, a concentration of flagellin of 100 ng/mL only induced a weak increase in TAP expression (3-fold) but failed to induce significant secretion of CXCL8 and increased expression of CCL20 (data not shown). Interestingly, response of PS cells to LPS required the addition of CD14 in the culture medium. Under serum-free conditions, PS cells responded only weakly to LPS. Nevertheless, CXCL8 secretion in response to 10 ng/mL LPS was increased in the presence of 5 μg/mL CD14 (1067 pg/mL) compared to only 67 pg/mL in the absence of CD14 (Figure 3E). On the contrary, supplementing the medium with LBP did not improve the response of PS cells to LPS. This indicates that CD14 is essential for the recognition of LPS by the PS cell line in our culture conditions. In the presence of serum (10% FBS), the PS cells produced CXCL8 in response to LPS, and CXCL8 production also occurred when the LPS stimulation was performed in fresh milk (Figure 3). This is in keeping with the occurrence of sCD14 in serum and bovine milk [30].

Compared to the response of primary cells to these same agonists, PS cells responded to a similar array of bacterial compounds, although their response was somewhat lower than that of pbMEC (Additional file 4). Furthermore, stimulation experiments performed at different passages indicated that the PS cell line is stable at least up to passage 24 (Additional file 4).

PS cells could thus be used as a surrogate to pbMEC to monitor the innate response of mammary epithelial cells and, in the present report, how this response can be modulated by IL-17A.

### IL-17A increases the response of PS cells to Pam3-CSK4

In a first attempt to investigate how IL-17A could modulate the response of MEC, we analysed the response of PS cells to purified bacterial agonists in the presence or not of IL-17A. We selected LPS and Pam3-CSK4, activating TLR4 and TLR1/2, respectively, as they are likely to represent the most relevant agonists in vivo through which *E. coli* stimulates MEC and they induce a strong innate response in pbMEC [16] and PS cells (see Figure 3).

**Figure 4:**
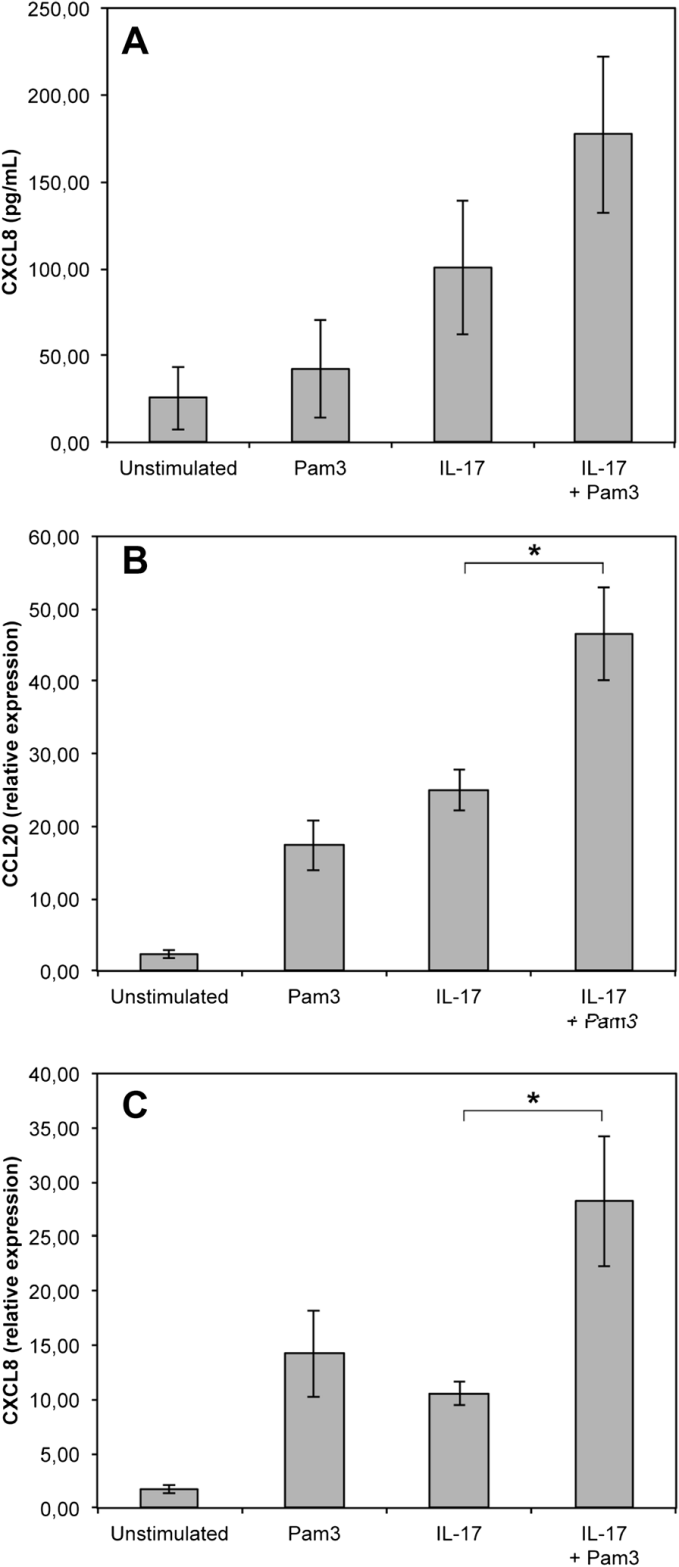
**IL-17A effect on the response of PS cells to Pam3-CSK4.** PS cells were incubated for 5 h with 100 ng/mL of Pam3-CSK4 in the presence or not of 100 ng/mL IL-17A. Response was analyzed in terms CXCL8 secretion (**A**) by ELISA or expression of CCL20 (**B**) and CXCL8 (**C**) by RT-qPCR. Data presented are mean values and SD obtained from 3 independent experiments. Experiments were performed at passages 15, 16 and 24. * statistical significance (*P* < 0.05) calculated using exact permutation tests after global comparison using a Kruskal and Wallis test.

**Figure 5:**
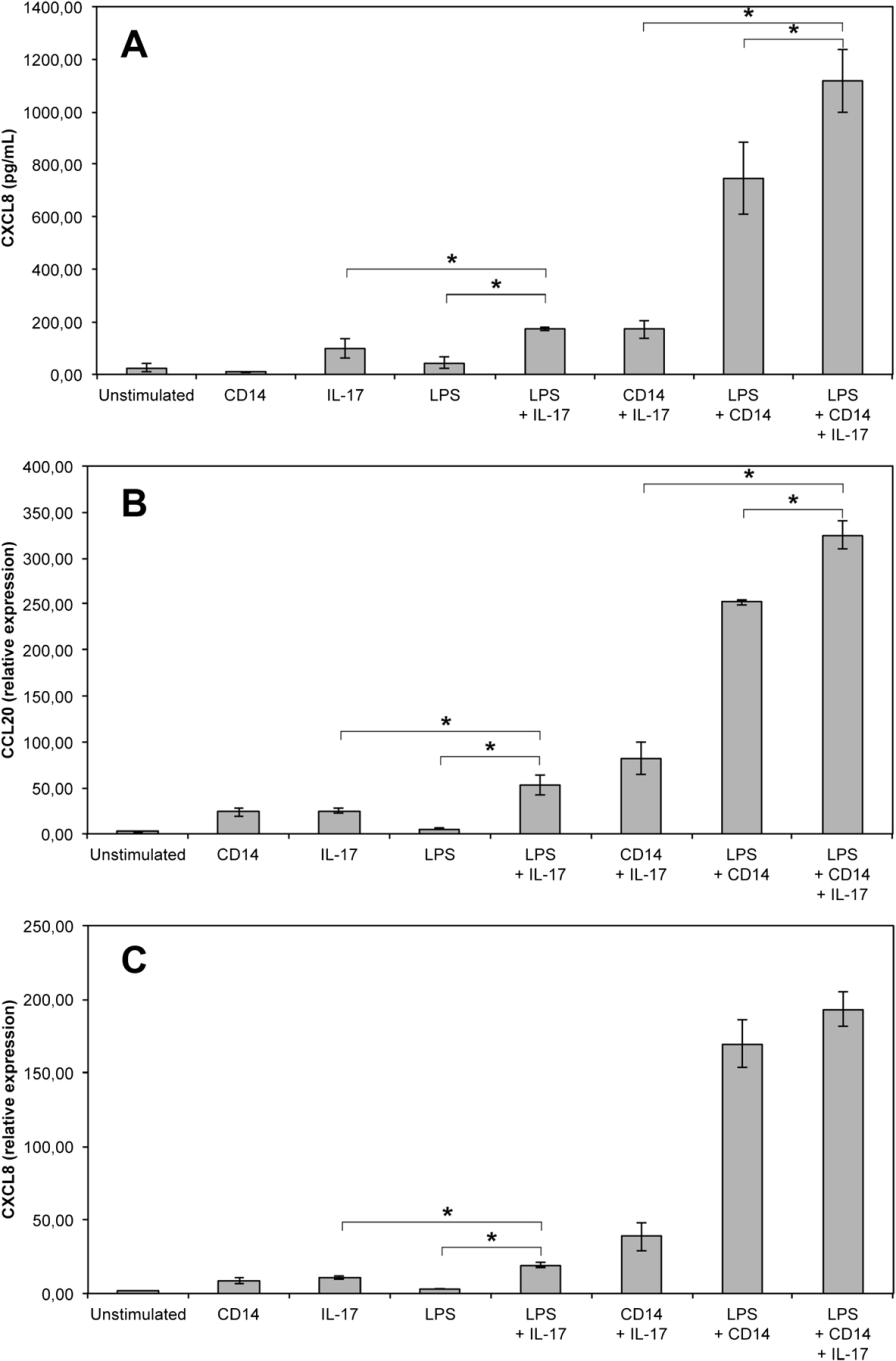
**IL-17A effect on the response of PS cells to LPS.** PS cells were incubated for 5 h with 10 ng/mL of LPS in the presence or not of 100 ng/mL IL-17A and 5 μg/mL CD14. Response was analyzed in terms CXCL8 secretion (**A**) by ELISA or expression of CCL20 (**B**) and CXCL8 (**C**) by RT-qPCR. Data presented are mean values and SD obtained from 3 independent experiments. Experiments were performed at passages 15, 16 and 24. * statistical significance (*P* < 0.05) calculated using exact permutation tests after global comparison using a Kruskal and Wallis test.

When PS cells were stimulated with both Pam3-CSK4 and IL-17 for 5 hours, the response in terms of CCL20 and CXCL8 gene expression was significantly increased compared to the response observed only in the presence of IL-17A or Pam3-CSK4 (Figure 4). The increase in terms of CXCL8 secretion, although significant statistically, was less important. The impact of IL-17A on the response of PS cells to LPS was also statistically significant but of lower magnitude. A clear response of PS cells to LPS was observed in the presence of CD14 with high level of secretion of CXCL8 and strong induction of CCL20 and CXCL8 gene expression (Figure 5). Secretion of CXCL8 was increased 1.5 fold when IL-17 was present along with LPS and CD14. In the same conditions, IL-17A induced only a small increase of CCL20 gene expression while no significant impact on CXCL8 gene expression was observed. Interestingly, although PS cells responded very weakly to LPS in the absence of CD14, we could observe that IL-17 increased slightly the response of PS cells when they were stimulated with LPS alone (Figure 5).

### IL-17A increases the response of MEC to live *E. coli*

We next analysed if IL-17A could also increase the response of MEC to live bacteria. We used a protocol previously described which allowed stimulation of cells with live bacteria while avoiding a change in pH of the culture medium due to bacterial outgrowth [21]: briefly, cells were incubated in the presence of bacteria for three hours. Bacteria were then washed away, medium containing gentamycin was added with or without IL-17A and incubation was prolonged for 5 h (for RT-qPCR analyses) or 21 h (for ELISA assays of CXCL8 secretion).

**Figure 6:**
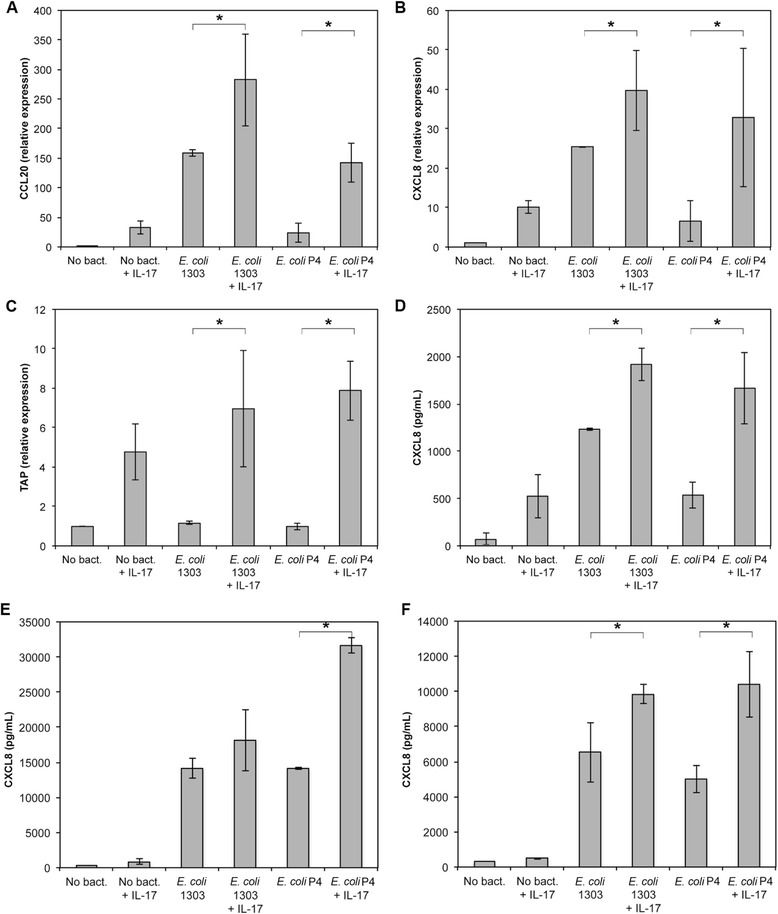
**IL-17A increases the secretion of CXCL8 by PS cells upon stimulation with** ***E. coli*** **strains 1303 and P4.** **A**-**D**: PS cells were incubated for 3 hours with strain *E. coli* 1303 or P4 at an MOI of 1. Cells were then washed twice with HBSS and medium with gentamycin was added with or without 100 ng/mL IL-17A. Response was analysed in terms of gene expression (CCL20: **A**, CXCL8: **B**, TAP: **C**) or CXCL8 secretion by ELISA (**D**) after beginning of the experiment. Data presented are mean values and SD obtained from 3 independent experiments. Experiments were performed at passages 8, 26 and 30. **E**-**F**: primary mammary epithelial cells from two cows were incubated for 3 hours with strains *E. coli* 1303 and P4 at an MOI of 1. Cells were then washed twice with HBSS and medium with gentamycin was added with or without 100 ng/mL IL-17A. CXCL8 secretion was measured by ELISA 24 hpi. Data presented are mean values obtained with cells stimulated in triplicates. * statistical significance (*P* < 0.05) calculated using exact permutation tests after global comparison using a Kruskal and Wallis test.

Results indicated that IL-17A increased the response of PS cells when they were stimulated with either *E. coli* strain 1303 or *E. coli* P4, a prototypical mastitis isolate. Expression of CCL20, CXCL8 and TAP were significantly increased upon addition of IL-17A in the culture medium (Figure 6). Similarly, secretion of CXCL8 was increased 1.5 fold and 3.1 -fold in PS cells stimulated with strains 1303 and P4, respectively (Figure 6D).

We observed a similar increase in innate response mediated by IL-17A using primary cells (Figures 6E-F). An increase in CXCL8 secretion was observed with primary cells from two different cows using *E. coli* P4 and, to a somewhat lower extent, with *E. coli* 1303. These results indicate that the increased pro-inflammatory response triggered by IL-17A is not restricted to PS cells. In addition, the fact that these primary cells do not require addition of CD14 in the medium to respond to LPS [16] suggests that the effect of IL-17A does not depend on supplementation of the medium with CD14.

Because synergies involving IL-17A have been described with IL-1β and TNFα we quantified IL-1β and TNFα in the supernatant of stimulated PS cells: amounts of IL-1β and TNFα were below the detection limit of the ELISA assays used (50 pg/mL for TNFα and 30 pg/mL for IL-1β).

## Discussion

The main objective of this report was to investigate if cytokines of the IL-17 family could play a role in the defence of cows against intramammary infections. This question is of particular relevance given the important role of IL-17 proteins in the defence of different hosts against a range of bacterial infections [28,31]. In addition, several reports indicate that IL-17 can be detected in bovine milk in different experimental settings. Production of IL-17A was recently demonstrated in the course of *S. uberis* infection [10]. Furthermore, vaccination of cows with a model antigen could induce a Th17 cell-mediated immune response characterized by the production, in particular, of IL-17A in the mammary gland [24]. The udder has therefore the ability to secrete IL-17A but its contribution to the defence against *E. coli* mastitis has not been investigated.

From the data presented in this report, we can clearly conclude that expression of IL-17A and IL-17F genes is induced approx. 100-fold upon infection by *E. coli*. We were however unable to detect any secretion of IL-17A in milk from *E. coli* infected quarters. So far, production of IL-17A in an infected udder was only reported in clinical mastitis after experimental *Streptococcus uberis* infection. Yet, IL-17A only reached significant levels after 56 h (peak level at 81 h), long after other cytokines had reached the peak level [10]. It is therefore possible that IL-17A levels could be detected in milk at later time points in the case of *E. coli* infections, explaining our failure to measure IL-17A in milk from *E. coli* infected cows 24 hours post-infection. Preliminary results indicates that expression of IL-17A and IL-17F genes is also induced in samples from quarters infected by the *S. aureus* strain 1027 (data not shown). Overall, expression of IL-17A and IL-17F genes during infection raise the point of the contribution of these cytokines to the defence of the udder against bacterial infection.

These cytokines are generally considered to act on epithelial cells as well as on endothelial and stromal cells by inducing, for example, expression of antimicrobial peptides and neutrophil-activating molecules [31]. We recently showed that, in vitro, IL-17A and IL-17F were likely to improve the innate defences of the host through modulation of gene expression patterns of MEC [13]. More precisely, primary MEC express IL-17A and IL-17F receptor genes and respond to the presence of IL-17A by increased expression of different pro-inflammatory mediators such as CXCL8, CCL20, IL-6 or IL-1β [13].

We therefore chose to investigate in more details if IL-17A could increase the response of the host to *E. coli* infection by modulating the pro-inflammatory response of MEC. To achieve this goal, we used a new cell line that we recently isolated. Prior to performing experiments with IL-17A, we characterized these cells in terms of expression of markers typical for MEC and in terms of response to purified compounds, as we had already done with primary cells [16].

The PS cell line was found to be stable, in terms of cell morphology (data not shown) and response to MAMPs, and displays a cobblestone aspect which is consistent with epithelia characteristics. The strong surface expression of CK18 and the low expression of CK14 allowed us to establish that PS cells are effectively from epithelial origin and of luminal nature [32-35]. The luminal character of PS cells is also indicated by the fact that PS cells formed unorganized clones and express PRRs [17,36]. The high percentage of CK18-positive cells, along with the lack of CD45 labelling, attested to the purity of this cell line and the absence of leukocytes. Additionally, these cells were shown to express α–casein gene CSN1S1, a proof of their secretory capacity, in accordance with their origin (i.e. secretory parenchyma) (E. Devinoy, M. Nguyen, personal communication).

Similar to pbMEC, PS cells express several PRRs, including TLR1, TLR2, TLR4, TLR6, NOD1 and NOD2 [16,25,36,37]. Consistent with the expression of TLR1, TLR2, TLR4 and TLR6, PS cells were able to respond to the presence of bacterial agonists that stimulates through these receptors such as the analogs of lipoproteins Pam 2-CSK4 and Pam 3-CSK4, and LPS provided CD14 is added in the culture medium.

Actually, the response of PS cells to LPS in the absence of CD14 was rather weak compared to what had already been described [16,37-39] and given the strong inflammation triggered by LPS infusion in the udder [40-42]. Supplementing our stimulation medium with purified CD14, or raw milk, resulted in a substantial increase of the immune response of PS cells to LPS. Others have reported similar findings with pbMEC [30]. These data suggest that the presence of CD14 in milk contributes to the response of MEC to LPS and concur with the observations that 1) concentrations of CD14 in milk are less than optimal and 2) that response of the host can be improved when CD14 is infused into the udder along with the *E. coli* inoculum [43].

As for flagellin, the absence of TLR5 transcript observed either with PS cells or primary MEC, along with the weak response induced by purified flagellin, are in agreement with previous in vitro and in vivo studies that suggest that flagellin is not a major contributor to the inflammatory response of the udder to *E. coli* infection [16,44].

The possibility that PS cells would de-differentiate after several passages, as was observed by Gunther et al. [15], was ruled out by showing that PS cells were still capable of inducing LAP expression upon stimulation with MAMPs.

Altogether, these results indicate that PS cells are useful surrogates to pbMEC in the understanding of innate immune response of MEC to MAMPs.

After having verified that PS cells expressed the genes encoding for the two sub-units of the IL-17A receptor, we used PS cells to investigate whether IL-17A could contribute to an increased innate immune response of MEC upon stimulation with purified bacterial compounds. Our results demonstrate that this could be the case as secretion of CXCL8, as well as expression of CCL20 and CXCL8 genes, are significantly increased when cells are incubated for 5 hours with IL-17A and purified Pam3-CSK4 or, to a lower extent, LPS. We were only able to detect an additive effect with no real synergy.

More interestingly, a synergy between IL-17A and innate response of MEC was observed when stimulation was performed with live bacteria. These results, initially obtained with PS cells, were confirmed with primary cells and extend the ones we obtained previously with purified bacterial compounds related to Gram-positive bacteria, i.e. MDP and LTA [13].

Our results are reminiscent to synergy observed with other mediators such as TNFα or IL-1β. [45-47]. Yet, we did not detect any IL-1β nor TNFα after stimulation of PS cells with live *E. coli*. It is therefore unlikely that production of these pro-inflammatory mediators could explain the synergies we observed. To explain such results, one should take into account the multiple activities of IL-17A both on induction of expression and on stabilization of mRNA transcripts.

The increased response of PS cells is most likely due to the stabilization of mRNA transcripts following IL-17A recognition as was observed for CXCL1 [48-50]. It could also be due to an increased expression of PRR genes such as TLR2 or TLR4. A third alternative is that expression of the IL-17A receptor at the surface of PS cells is increased after infection by *E. coli*. Yet, in the absence of suitable reagent, such a possibility cannot be tested. The origin of the synergy observed will have to be further investigated.

Another major issue concerns the characterization of the cells that overexpress the genes for IL-17A and IL-17F upon *E. coli* infection. Immunocytochemistry analyses suggested that MEC or interstitial cells, presumably lymphocytes, could produce IL-17A upon inflammatory challenge [24]. Interestingly, one of the genes assayed in our work, CCL20, encodes a ligand for the CCR6 receptor which is expressed by effector memory T cells, including Th17 cells [51]. As a consequence, the increased expression of CCL20 that we detected in our assays could also participate to the amplification of the host response to infection by attracting more IL-17-producing T cells to the site of infection.

In conclusion, our results show a marked overexpression of the genes encoding IL-17A and IL-17F in the mammary gland during *E. coli* infections which, by extending previous observation of overexpression during *S. uberis* udder infections, strongly suggest that these cytokines and the cells that produce them are important players of host-pathogen interactions during mastitis. Most interestingly, we were able to demonstrate that a potential role for IL-17A is to increase the pro-inflammatory and antimicrobial responses of MEC after *E. coli* infection. These results are also interesting in the scope of vaccine development. As a matter of fact, the protocol used in the present manuscript using live bacteria mimics the in vivo situation encountered by the bacteria entering the udder of vaccinated animals. In such cases, it is expected that the first response of MEC occurs in the absence of IL-17A, IL-17A coming into play only in a second step when memory Th17 cells have been activated. Our results are thus a strong incentive to further investigate the contribution of IL-17A to the defence of the mammary gland.

## Additional files

Additional file 1:
**Antibodies list.** List of antibodies used in this study. Additional file 2:
**Primer table.** List of primers used in this study. Additional file 3:
**Microscopic examination of PS cells.** A, PS cells were photographed under phase contrast (100x). B, PS cells after May Grünwald-Giemsa straining (200x). Additional file 4:
**Innate immune response of PS cells and pbMEC to different purified bacterial agonists.** pbMEC from two cows (white bars) and PS cells at passages 10 and 24 (grey bars) were incubated for 5 h with the indicated concentrations of different purified agonists. Response was analyzed in terms of expression of CCL20, TAP, LAP and CXCL8 genes by RT-qPCR. Data are mean values of stimulations performed in duplicates.
