# Supplementary material for: Investigating the contribution of IL-17A and IL-17F to the host response during Escherichia coli mastitis
Source: Vet Res. 2015 Jun 11;46(1):56. doi: 10.1186/s13567-015-0201-4 (PMC4462179; doi:10.1186/s13567-015-0201-4)
Supplement: Additional file 1: — Antibodies list. List of antibodies used in this study. [file 13567_2015_201_MOESM1_ESM.docx]

**Additional file 1 List of antibodies used in this study.**

| Antigen | Primary antibody | Source | Dilution | Secondary antibody | Source | Dilution |
| --- | --- | --- | --- | --- | --- | --- |
| CK18 | Chicken polyclonal, ab14047 | Abcam, Cambridge, UK | 1/100 | FITC-conjugated Rabbit anti-chicken, ab6749 | Abcam, Cambridge, UK | 1/100 |
| CK14 | Mouse monoclonal anti human (clone LL002) | AbD Serotec, Oxford, UK | 1/50 | RPE-conjugated Rabbit F(ab’)2 anti-mouse IgG | AbD Serotec, Oxford, UK | 1/50 |
| CD24 | PE-conjugated rat monoclonal anti mouse (clone M1/69) | BD Biosciences | 1/30 |  |  |  |
| CD45 | Mouse monoclonal anti bovine (clone CACTB51A) | VMRD, Pullman USA | 1/100 | FITC conjugated swine anti-mouse | Nordic Immunology, Tilburg, The Netherlands | 1/80 |
| CD14 | Alexafluor647-conjugated mouse monoclonal anti-human (clone Tük4) | AbD Serotec, Oxford, UK | 1/30 |  |  |  |
| CD282 (TLR2) | Alexafluor647-conjugated human monoclonal anti-bovine (clone 12538) | AbD Serotec, Oxford, UK | 1/10 |  |  |  |
