# Supplementary material for: Investigating the contribution of IL-17A and IL-17F to the host response during Escherichia coli mastitis
Source: Vet Res. 2015 Jun 11;46(1):56. doi: 10.1186/s13567-015-0201-4 (PMC4462179; doi:10.1186/s13567-015-0201-4)
Supplement: Additional file 2: — Primer table. List of primers used in this study. [file 13567_2015_201_MOESM2_ESM.docx]

**Additional file 2 PCR primers used in this study.**

| Target gene | Oligonucleotides (5'-3') | Product size | Annealing temperature | Target sequence (Ref NCBI) |
| --- | --- | --- | --- | --- |
| 18S | CGGGGAGGTAGTGACGAAA | 196pb | 62 °C | AF176811 |
|  | CCGCTCCCAAGATCCAACTA |  |  |  |
| ACTB | ACGGGCAGGTCATCACCATC | 166pb | 65 °C | BT030480 |
|  | AGCACCGTGTTGGCGTAGAG |  |  |  |
| PPIA | TCCGGGATTTATGTGCCAGGG | 206pb | 65 °C | BC105173 |
|  | GCTTGCCATCCAACCACTCAG |  |  |  |
| TLR1 | ACCCTACTCTGAACCTCAAG | 142pb | 62 °C | NM_001046504.1 |
|  | GACTGCACACTGGATTTCTG |  |  |  |
| TLR2 | ACTGGGTGGAGAACCTCATGGTCC | 307pb | 62 °C | NM_144197.2 |
|  | ATCTTCCGCAGCTTACAGAAGC |  |  |  |
| TLR4 | GCATGGAGCTGAATCTCTAC | 238pb | 62 °C | NM_174198.6 |
|  | CAGGCTAAACTCTGGATAGG |  |  |  |
| TLR5 | TTCCTGCAACCTCACCCAAG | 192pb | 62 °C | NM_001040501.1 |
|  | CTGAGATTGGGCAGGTTTCG |  |  |  |
| TLR6 | CTCCGGGAGATAGTCACTTC | 297pb | 62 °C | NM_001001159 |
|  | GGCCCTGGATTCTATTATGG |  |  |  |
| NOD1 | TGGTCACTCACATCCGAAAC | 218pb | 62 °C | XM_598513 |
|  | AGGCCTGAGATCCACATAAG |  |  |  |
| NOD2 | CCCAGGGGCTCAGAACTAACA | 238pb | 62 °C | NM_001002889 |
|  | CCTTCATCCTGGACGTGGTTC |  |  |  |
| MD2 | AATCGTTGGGTCTGCAACTC | 210pb | 62 °C | NM_001046517.1 |
|  | GCGCAATGGGAAATTCATGG |  |  |  |
| CCL20 | TTCGACTGCTGTCTCCGATA | 172pb | 62 °C | NM_174263 |
|  | GCACAACTTGTTTCACCCACT |  |  |  |
| CXCL8 | TGAAGCTGCAGTTCTGTCAAG | 202pb | 62 °C | NM_173925.2 |
|  | TTCTGCACCCACTTTTCCTTGG |  |  |  |
| LAP | TGCTCCTTGCGCTCCTCTTC | 149pb | 62 °C | NM_203435 |
|  | CTCCGAGACAGGTGCCAATC |  |  |  |
| TAP | GTAGGAAATCCTGTAAGCTGTG | 139pb | 62 °C | AF014106 |
|  | GTGTCTTGGCCTTCTTTTAC |  |  |  |
| IL-17RA | GGCTGAACTGCACAGTCAAG | 159pb | 62 °C | XP_603383 |
|  | AGCGTCCACTCGATGTGAAC |  |  |  |
| IL-17RC | TGCCCTGGTTCCTTCTGTCC | 139pb | 62 °C | BC122679 |
|  | AGGCAGAGCACGTCACCATC |  |  |  |
| IL17C | TGACGTCCACCAGCGCTCCATC | 163pb | 62 °C | ENSBTAT00000065977 |
|  | CTGGACCAGCGGCACTGAGTTG |  |  |  |
| IL17E | ACTGAGGAGTGGCTGAAGTG | 161 pb | 64 °C | ENSBTAT00000008806 |
|  | AGCCGGTTCAAGTCTCTGTC |  |  |  |
