# Supplementary figures and images for: Investigating the contribution of IL-17A and IL-17F to the host response during Escherichia coli mastitis
Source: Vet Res. 2015 Jun 11;46(1):56. doi: 10.1186/s13567-015-0201-4 (PMC4462179; doi:10.1186/s13567-015-0201-4)

**A**

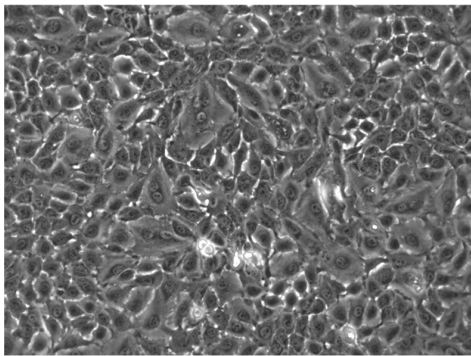

**B**

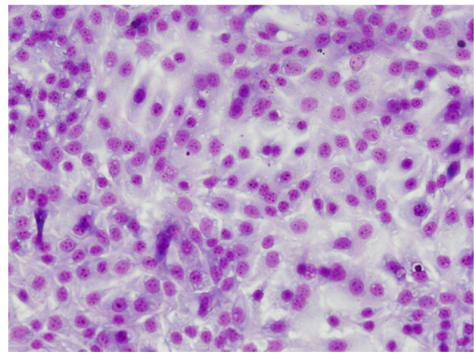

Supplement: Additional file 3: — Microscopic examination of PS cells. A, PS cells were photographed under phase contrast (100x). B, PS cells after May Grünwald-Giemsa straining (200x). [file 13567_2015_201_MOESM3_ESM.pdf]

**A**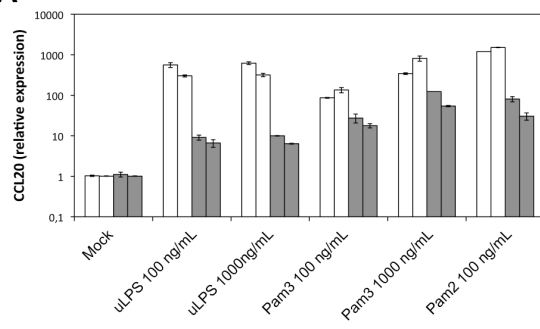**B**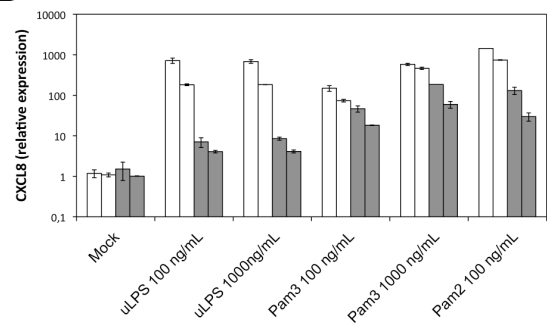**C**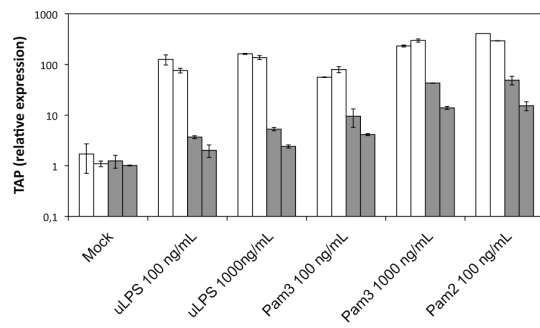**D**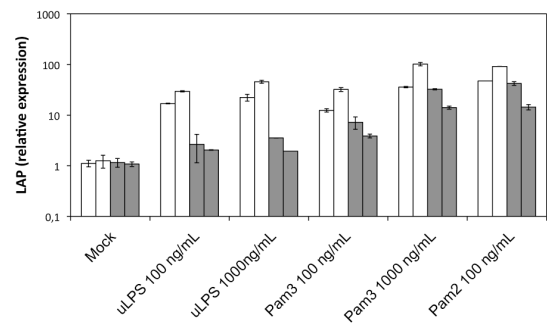

Supplement: Additional file 4: — Innate immune response of PS cells and pbMEC to different purified bacterial agonists. pbMEC from two cows (white bars) and PS cells at passages 10 and 24 (grey bars) were incubated for 5 h with the indicated concentrations of different purified agonists. Response was analyzed in terms of expression of CCL20, TAP, LAP and CXCL8 genes by RT-qPCR. Data are mean values of stimulations performed in duplicates. [file 13567_2015_201_MOESM4_ESM.pdf]
